# Cardiac fibroblasts secrete exosome microRNA to suppress cardiomyocyte pyroptosis in myocardial ischemia/reperfusion injury

**DOI:** 10.1007/s11010-021-04343-7

**Published:** 2022-02-04

**Authors:** Niannian Liu, Liang Xie, Pingxi Xiao, Xing Chen, Wenjie Kong, Qiaozhen Lou, Feng Chen, Xiang Lu

**Affiliations:** 1 Department of Cardiology, The Fourth Affiliated Hospital of Nanjing Medical University, Nanjing, 210031 China; 2 Department of Cardiology, Jinling Hospital, Nanjing University School of Medicine, Nanjing, 210000 Jiangsu China; 3 Department of Geriatrics, The Affiliated Sir Run Run Hospital of Nanjing Medical University, Nanjing, 211166 China; 4 Department of Forensic Medicine, Nanjing Medical University, No. 101 Longmian Road, Jiangning District, Nanjing, 211166 Jiangsu China

**Keywords:** microRNA, Exosome, Myocardial ischemia/reperfusion injury, Sudden cardiac death

## Abstract

Molecular mechanisms underlying myocardial ischemia/reperfusion (MI/R) injury and effective strategies to treat MI/R injury are both in shortage. Although pyroptosis of cardiomyocytes and the protective role of cardiac fibroblasts (CFs) have been well recognized as targets to reduce MI/R injury and sudden cardiac death (SCD), the connection has not yet been established. Here, we showed that CFs protected cardiomyocytes against MI/R-induced injury through suppression of pyroptosis. A novel molecular mechanism underpinning this effect was further identified. Under hypoxia/reoxygenation condition, CFs were found to secrete exosomes, which contain increased level of microRNA-133a (miR-133a). These exosomes then delivered miR-133a into cardiomyocytes to target ELAVL1 and repressed cardiomyocyte pyroptosis. Based on this finding, we successfully developed a new strategy that used exosomes derived from CFs with overexpressed miR-133a to enhance the therapeutic outcomes for the MI/R injury. Overall, our results provide a novel molecular basis for understanding and treating MI/R injury, and our study also provides novel insight for the postmortem diagnosis of MI/R injury induced SCD by using exosome biomarker in forensic.

## Introduction

Myocardial ischemia/reperfusion (MI/R) injury is now a well-known pathological incident, which can lead to acute myocardial infarction, heart failure and even sudden cardiac death (SCD) [1]. It is worth mentioning that acute myocardial infarction is one of the common causes which increase the morbidity and mortality in the population worldwide [2]. In case of acute myocardial infarction, unexpected occlusion of coronary artery which leads to cardiomyocyte damage is supposed to occur at the first stage [3]. Therefore, reperfusion is critical to save myocardium, however restoration of blood flow to the ischemic myocardium paradoxically causes injury at the second stage. The whole process is deemed as MI/R injury, which becomes a hot topic in the field of treatment of ischemia heart diseases. The molecular mechanisms underlying MI/R injury include oxidative stress, inflammation, mitochondrial dysfunction, and cell death [4, 5]. Several strategies for battling MI/R injury and reducing myocardial infarction size, such as ischemic conditioning, pharmacological treatment and physical intervention, have also been developed [6]. However, the molecular mechanisms of MI/R injury are still elusive and the translation of above mentioned cardioprotective strategies is also far from satisfactory. Therefore, further exploration of molecular events during MI/R injury to advance therapeutic development is avidly required.

It has become clear that cardiomyocyte death has determinant roles in both MI/R injury, heart failure and SCD [7]. Different forms of cell death, such as necrosis, apoptosis and pyroptosis, are involved in MI/R injury. Pyroptosis is a form of programmed necrosis but different from other programmed cell deaths, is found to be extensively implicated in MI/R injury [8]. Pyroptotic cells are characterized by bubbling and swelling of the cell membrane until the cell ruptures [9]. Specifically, the activation of caspase-1 cleaves gasdermin D (GSDMD) to generate N-terminal fragment oligomers in the cell membrane, which leads to pyroptotic cell death through the formation of large pore [9, 10]. It has been discovered that, during cardiomyocyte pyroptosis in MI/R injury, NOD-like receptor protein 3 (NLRP3) inflammasome is activated in cardiomyocyte, eventually leading to the activation of caspase-1 and release of inflammasome-related cytokines interleukin-1β (IL-1β) and interleukin-18 (IL-18) [11]. Moreover, pyroptosis mediated by NLRP3 activation is also able to further aggravate MI/R injury [12, 13]. Hence, targeting pyroptosis signaling pathways to reverse this process has been considered as a promising therapeutic approach against MI/R injury [8, 14]. Detailed elucidation of the molecular events underlying cardiomyocyte pyroptosis in MI/R injury to guide therapeutic intervention thus deserves exploration.

In myocardium, there exist multiple cell types, which can execute cardioprotective function. As the largest population of cells in myocardium, cardiac fibroblasts (CFs) are known to play pivotal roles in cardiac development and maintaining cardiac function [15]. It is worth noting that the protective roles of CFs in MI/R injury have recently been demonstrated, showing that CFs can significantly increase the viability of cardiomyocytes and decrease myocardial infarct size [16]. Molecular mechanisms underpinning the cardioprotective function of CFs have been attributed to intercellular communication between them and cardiomyocytes [17]. Furthermore, extracellular vesicles including exosomes and microvesicles have been identified as key intermediates for the intercellular communication [16, 18]. Extracellular vesicles secreted by donor cells can carry diverse functional biomolecules and deliver them into recipient cells, resulting in execution of function of carried biomolecules in recipient cells [16]. For example, it has been found that CFs derived exosomes carrying functional microRNAs (miRNAs) or proteins into cardiomyocytes increase cardiomyocyte survival and protect against MI/R injury [17, 18]. Nevertheless, studies regarding to whether and how CFs derived exosomes to avert cardiomyocyte pyroptosis and thus exert cardioprotective function have largely lagged behind.

The present study aimed to explore whether CFs derived exosomes protect cardiomyocytes against MI/R injury through suppressing cardiomyocyte pyroptosis, and to further investigate the potential molecular mechanisms in MI/R-induced injury in order to provide a novel approach for effective treatment of MI/R injury.

## Materials and methods

### Cell culture and establishment of hypoxia/reoxygenation (H/R) injury model

Rat myocardial cell line H9C2 and 293 T cells were purchased from Cell Resource Center of Shanghai Academy of Sciences, and myocardial fibroblasts were isolated from neonatal mice as described previously [19]. H9C2 cells, 293 T cells, and CFs were cultured in DMEM (Gibco, Grand island, NY, USA) with 10% (v/v) fetal bovine serum (Gibco), and 1% (v/v) antibiotics (100 units/ml penicillin and 100 µg/ml streptomycin (MP Biomedicals, Santa Ana, CA, USA). The cells were maintained in a humidified incubator containing 5% CO_2_ at 37℃. When H9C2 cells reached 60–70% confluency, DMEM medium was replaced with a serum-free and sugar-free medium, and then the cells were exposed to hypoxia conditions with 1% O_2_, 94% N_2_, and 5% CO_2_ for 6 h. Then, the medium was replaced by fresh medium and nursery was refilled with air containing 5% CO_2_ for the indicated time to establish the cardiomyocyte model of H/R injury. Lipopolysaccharide (LPS, Sigma-Aldrich) was dissolved in culture medium and used at concentration of 0.1 µg/ml or 1 µg/ml, and H9C2 cells were exposed to culture medium along with LPS as a positive control.

### Cell co-culture

Transwell chambers (Corning Inc., Corning, NY, USA) containing 6.5 mm-diameter polycarbonate filter (1 μm pore) were used to co-culture of CFs and H9C2 cells, CFs were seeded in the upper compartments of cell culture transwell, while H9C2 cells were cultured on the lower compartments. H9C2 cells were collected 2 days after co-culture. Before the co-culture experiments, H9C2 cells were treated by hypoxia-reoxygenation. Exosome inhibitor GW4869 (Sigma-Aldrich) was used to treat cultured CFs in the upper compartments.

### Exosome isolation

When cells reached 70–80% confluency, culture medium was replaced with that containing 5% exosome-depleted fetal bovine serum (System Biosciences, Palo Alto, CA, USA) and cultured for 48 h. Exosomes were isolated using differential centrifugation as reported before [20]. Briefly, cell culture supernatant was collected after 48 h with exosome-depleted medium, and centrifuged twice at 500×*g* for 10 min at 4 °C, and then centrifuged once at 2000×*g* for 20 min and 10,000×*g* for 30 min at 4 °C to remove debris. The supernatant was centrifuged at 100,000×*g* for 90 min to pellet exosomes. Further, exosomes were then washed and resuspended in phosphate buffer saline (PBS, Gibco) and centrifuged at 10,000×*g* for 90 min, and purified by sucrose-gradient centrifugation.

### Transmission electron microscope (TEM)

After isolation, the exosome samples were diluted with PBS (Gibco) and then applied to 200-mesh nickel grids for fixation. Samples were stained with 2% phosphotungstic acid (Sigma-Aldrich) for 5 min at room temperature, and air-dried. Exosomes were detected through the transmission electron microscope (Hitachi, Tokyo, Japan) at 80 kV.

### Dynamic light scattering (DLS)

The detection of exosomes was performed as previously reported [21]. Briefly, 10 µl aliquot from purified and resuspended exosome was diluted in 990 µl PBS (Gibco), mixed well and loaded into cuvettes. Exosome sample volume of 1 ml was measured and three independent readings were performed. High Performance Particle Sizer (Malvern, Worcestershire, UK) was used in this series of experiments, and data acquisition and analysis were performed using Dispersion Technology Software configured for HPPS analysis.

### Exosome transfer and co-culture

Exosomes from CFs were stained by green fluorescent linker PKH67 (Sigma-Aldrich, St. Louis, MO, USA) in accordance with manufacture’s protocols. H9C2 cells were inoculated into 24-well plates at the density of 5 × 10^5^ cells per well. Cells were incubated by culture medium containing PKH67 labeled exosomes (20 μg/ml) for 24 h. Then, the samples were observed and photographed under the fluorescence microscope (Carl Zeiss, Jena, Germany).

### Cell viability assay

Cell viability was determined using Cell Counting Kit-8 (CCK-8, Synthgene, Nanjing, Jiangsu, China). In brief, cells were plated in 96-well plates (1 × 10^3^ cells per well) and cultured overnight. The cells were then treated with 10 µl of CCK-8 reagent and cultured at 37 °C for 2 h. Absorbance at 450 nm of each sample was recorded.

### RNA isolation and real-time quantitative PCR (RT-qPCR)

Total RNA was isolated from cultured cells and purified exosome using TRIzol® reagent (Thermo Fisher Scientific, Waltham, MA, USA) and RNA was reverse transcribed to cDNA by using PrimeScript RT reagent Kit with gDNA Eraser (Takara Bio, Kusatsu, Japan) according to the manufacturer’s protocol. RT-qPCR was performed by using SYBR Premix Ex Taq (Takara Bio) according to the manufacturer’s protocol on an Applied Biosystems 7300 sequence detection system (Thermo Fisher Scientific). U6 levels were used to normalize the relative abundance of miRNAs, and GAPDH was used to normalize the expression of ELAVL1, NLRP3, and caspase-1.

### Western blot

H9C2 cells or rat myocardial tissues were harvested, and protein was extracted using RIPA lysis buffer (Thermo Fisher Scientific) that includes protease inhibitors (Roche Diagnostic, Indianapolis, IN, USA). The protein extraction process was kept on ice. The quantity of total protein was determined by BCA assay (Thermo Scientific). Protein samples (20 µg) were  loaded  and fractionated by 10% SDS-PAGE and then transferred to polyvinylidene difluoride membrane (Millipore, Billerica, MA, USA). Subsequently, membranes were incubated with 5% non-fat milk containing 0.1% PBST to block nonspecific binding for 2 h at room temperature and treated with primary antibodies against ELAVL1 (1:1000, Abcam, Cambridge, MA, USA), NLRP3 (1:1000, Abcam), cleaved caspase-1 (1:1000, Cell Signaling Technology, Danvers, MA, USA), and GAPDH (1:1000, Abcam) at 4℃ overnight, respectively, followed by incubation with the relevant horseradish peroxidase (HRP) conjugated secondary antibody (1:5000, Abcam) at room temperature for 2 h. The protein bands were visualized using an enhanced chemiluminescence kit (Synthgene) and quantified by analysis using the ImageJ software.

Exosome marker proteins CD9, CD63, and TSG101 were identified by western blot, the protocol being similar to that used for cell and tissue protein detection.

### Enzyme linked immunosorbent assay (ELISA)

Rat blood and cell culture medium were collected, respectively, and then serum or cell culture supernatant was obtained through stand procedure with 825×*g* centrifuge for 10 min at 4 ℃. The activities of specific markers including IL-1β and IL-18 were determined according to the manufacturer's instructions (R&D Systems, Santa Clara, CA, USA).

### Cell transfection

The CFs or H9C2 cells were seeded in 6-well plates. MiR-133a mimic, mimic control, miR-133a inhibitor or inhibitor negative control (Synthgene) were transfected into H9C2 cells. MiR-133a mimic or mimic control were transfected into CFs. When the confluence of cells was up to 60%, the transfection was performed using Lipofectamine 2000 (Thermo Fisher Scientific) according to the manufacturer's instructions.

### Luciferase reporter assay

The entire 3′-UTR of ELAVL1 containing the predicted binding sites for miR-133a and the binding sequences mutant ELAVL1 3′-UTR was amplified and inserted into a luciferase reporter plasmid (Promega, Madison, WI, USA). For the luciferase reporter assay, cells were plated in 24-well plates, and each well was transfected with 1 µg of luciferase reporter plasmid, 1 µg of β-galactosidase plasmid (internal control), and 100 pmol of miR-133a mimic or control mimic using Lipofectamine 2000 (Thermo Fisher Scientific). After 48 h, luciferase signals were measured using a luciferase assay kit according to the manufacture’s protocol (Promega).

### Animals model

Male Sprague–Dawley rats (8–10 w and ca. 220 g weight) were obtained from Nanjing Biomedical Research Institute of Nanjing University. All animals were raised according to the standard procedure, and experiments were performed in an ethical manner, being approved by the ethics committee of Nanjing Medical University. Following acclimatization at least 1 week, the rats were randomly divided into four groups before the operation: sham, I/R, I/R added exosomes, I/R added exosomes from overexpressed miR-133a CFs. The methods of myocardial I/R model building refer to previous articles [22]. Rats were anesthetized by use of sodium pentobarbital (45 mg/kg, ip), and subsequently, the left coronary artery (LCA) was exposed using left thoracotomy at the fifth intercostal space. Following LCA ligation with 7–0 silk sutures, a smooth catheter was introduced and advanced down the artery to achieve ischemia for 30 min, and then rats were sacrificed after reperfusion for 120 min. The sham group rats underwent similar surgery, but without the LCA I/R, and were treated with saline. In other groups, rats were injected with PBS or same volume PBS that containing 200 μg exosome into myocardium after I/R injury.

### Hematoxylin and eosin (HE) staining

After we excised the myocardial tissues from the animals, tissues were fixed with 4% paraformaldehyde (Thermo Fisher Scientific) for 24 h and then paraffin-embedded. Sections of 4 µm were cut and staining was carried out according to the protocol of HE staining (Beyotime Biotechnology, Beijing, China).

### Tunel detection

The hearts of rats were isolated and fixed in 4% paraformaldehyde (Thermo Fisher Scientific) and were then embedded in paraffin, and TUNEL staining was processed using a Colorimetric TUNEL Apoptosis Assay Kit (Beyotime Biotechnology) according to the manufacturer's instructions. All nuclei were stained by DAPI. TUNEL-positive staining patterns were acquired using optical microscope (Olympus, Tokyo, Japan) with a 10× or 40× objective.

### Statistical analysis

All data were expressed as the mean ± standard deviation (SD) of three independent experiments. Student’s t-test was used for comparison between two groups, and one-way ANOVA was performed to test the mean difference between multiple groups. All analysis was carried out by GraphPad Prism 5.0 (GraphPad Software Inc., La Jolla, CA, USA) and *p* value < 0.05 was considered statistically significant.

## Results

### Pyroptosis of cardiomyocytes is involved in H/R injury

**Fig. 1:**
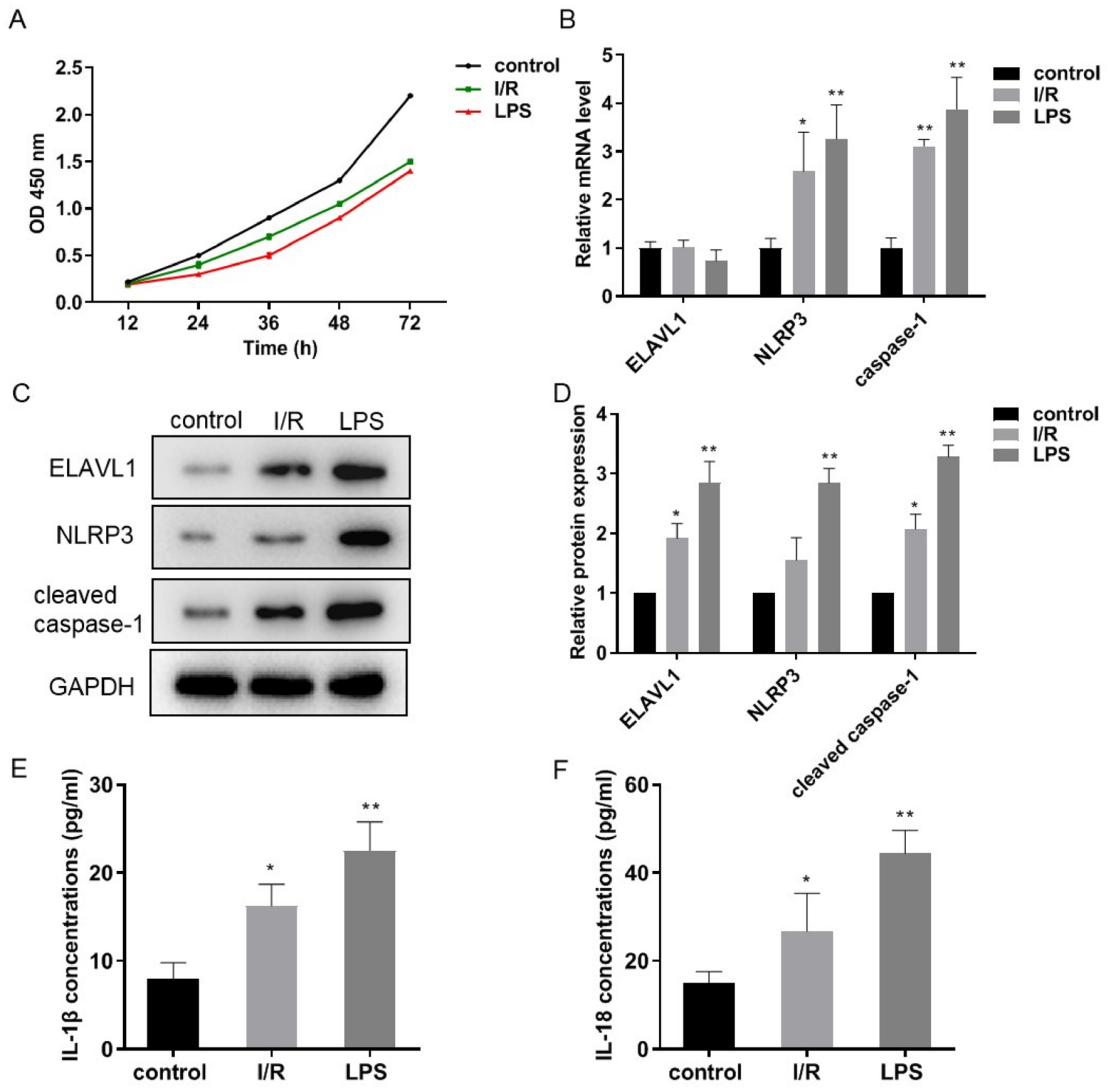
Pyroptosis of cardiomyocytes is involved in H/R injury. **A** Cell viability in H/R cardiomyocytes was measured by CCK8 kit, the time points indicate the reoxygenation time course after 6 h hypoxia. **B** RT-qPCR assayed ELAVL1, NLRP3, caspase-1 mRNA expression levels in H/R cardiomyocytes with 6 h hypoxia/1h reoxygenation. **C** and **D** Western blot detected the protein levels of ELAVL1, NLRP3, and cleaved caspase-1 in H/R cardiomyocytes. **E** and **F** The IL-1β and IL-18 expression levels were determined by ELISA kit. LPS was used as a positive control. *n* = 3, data are shown as mean ± SD. *p < 0.05, **p < 0.01 *vs*. the control group

Cell pyroptosis was induced in cardiomyocytes treated with hypoxia followed by reoxygenation with oxygenated solution to mimic MI/R-induced injury. LPS was used as a positive control. As shown in Fig. 1A, in comparison with control group without treatment, a significant cardiomyocyte death was observed in H/R group and LPS group. We next measured expression levels of prominent makers for H/R injury and pyroptosis, including ELAV-like RNA-binding protein 1 (ELAVL1) [23], NLRP3, and caspase-1 in cardiomyocytes. As shown in Fig. 1B–D, the mRNA and protein levels of NLRP3 and caspase-1 were upregulated in H/R and LPS groups. The mRNA level of ELAVL1 remained unchanged, but its protein level was significantly increased in H/R and LPS groups. We also measured inflammatory cytokines IL-1β and IL-18 secreted by cardiomyocytes. As expected, the expression levels of IL-1β and IL-18 in the culture medium were obviously increased in H/R and LPS groups (Fig. 1E, F). These data suggest that pyroptosis of cardiomyocytes is exerted in H/R injury.

### CFs avert pyroptosis of cardiomyocytes

**Fig. 2:**
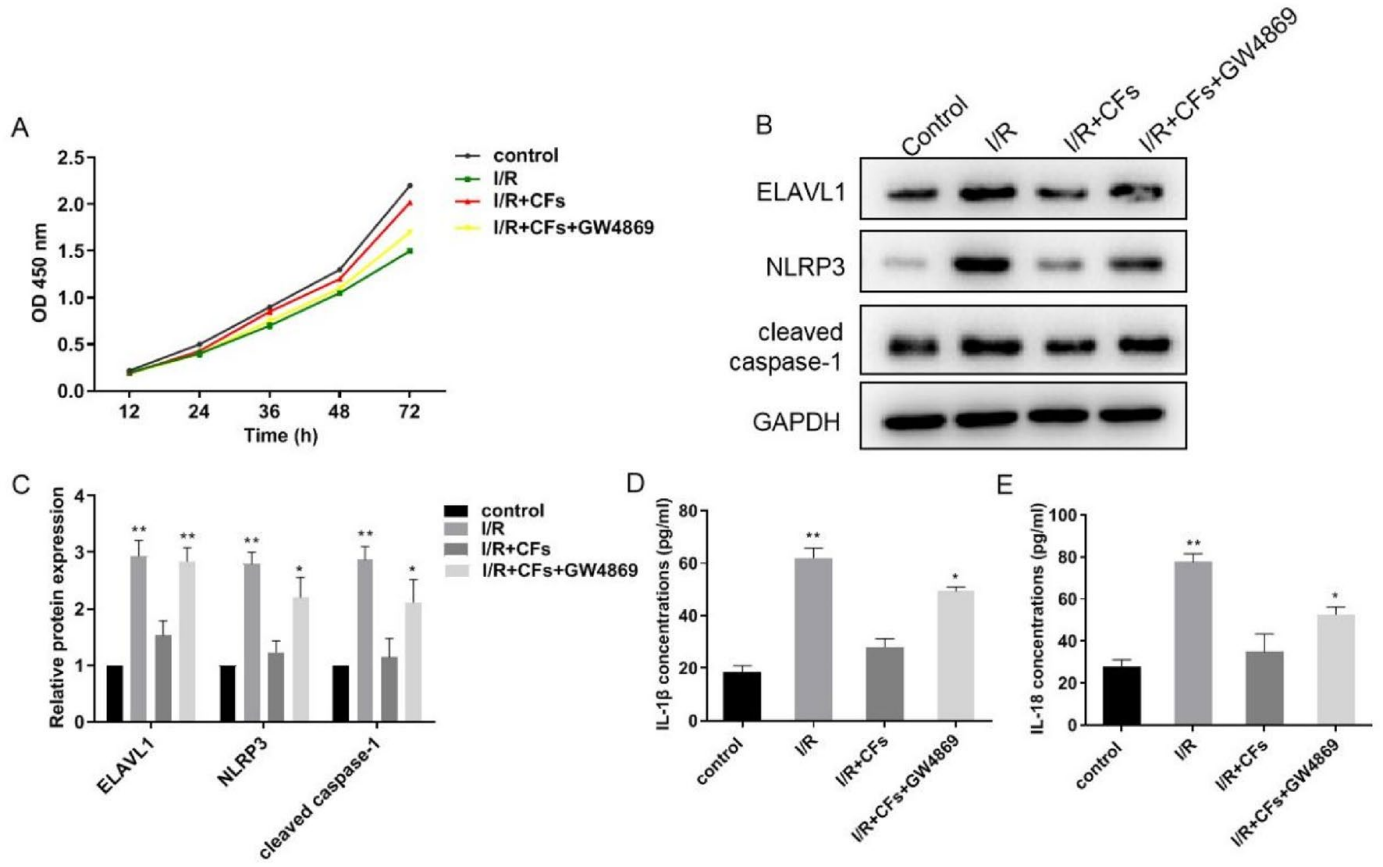
CFs avert pyroptosis of cardiomyocytes. H/R cardiomyocytes co-culture with CFs, GW4869 (10 uM) was used to inhibit the exosome secretion from CFs. **A** CCK8 kit was used to measure the cell viability. **B** and **C** Protein levels of ELAVL1, NLRP3, and cleaved caspase-1 were detected by western blot and quantitative analysis of the protein expression. **D** and **E** IL-1β and IL-18 expression was determined by ELISA kit. *n* = 3, data are shown as mean ± SD. *p < 0.05, **p < 0.01 *vs*. the control group

Next, we investigated whether CFs can avert pyroptosis, a well-recognized phenomenon of MI/R injury which can further aggravate MI/R injury. Excitingly, upon incubation with CFs, cardiomyocytes treated with H/R underwent significantly lowered cell death (Fig. 2A). We then checked the expression levels of pyroptosis markers in cardiomyocytes and inflammatory cytokines released by cardiomyocytes. As shown in Fig. 2B and C, co-incubation of CFs obviously decreased protein levels of ELAVL1, NLRP3, and caspase-1 in cardiomyocytes treated with H/R. Similarly, IL-1β and IL-18 levels were decreased by CFs (Fig. 2D, E). Since previous reports have indicated that CFs can exert cardioprotective function through secreting exosomes [24], we used GW4869 to inhibit the secretion of exosomes by CFs. As shown in Fig. 2A, the treatment of GW4869 diminished the suppressive effect of CFs on pyroptosis in cardiomyocytes. Consistently, the suppression of the expression levels of pyroptosis markers and inflammatory cytokines were reversed by treatment with GW4869 (Fig. 2B–E). Taken together, CFs can avert pyroptosis of cardiomyocytes induced by H/R and this protective effect depends on exosomes secreted by CFs.

### Exosomes secreted by CFs reduce pyroptosis of cardiomyocytes

**Fig. 3:**
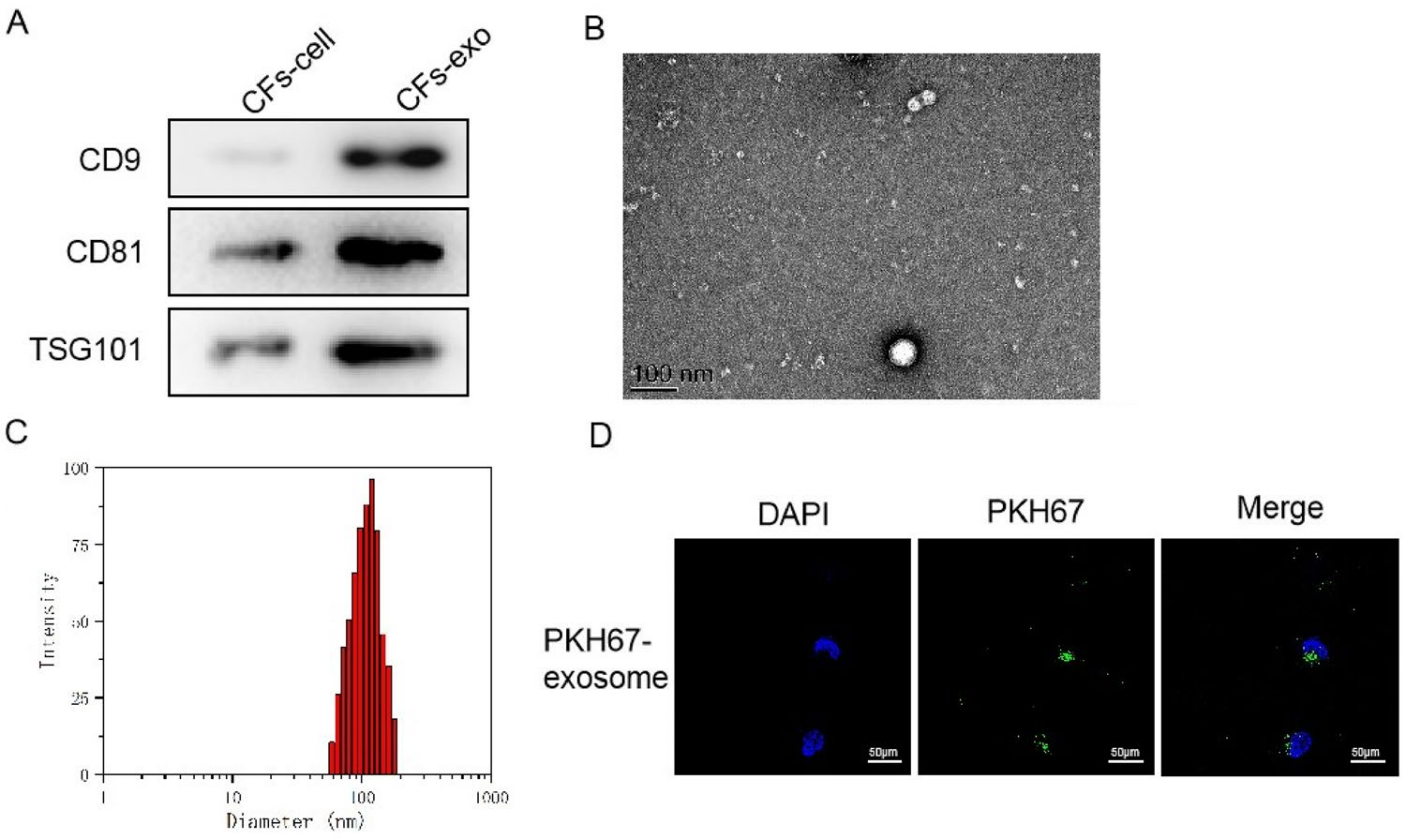
Characterization of CF-derived exosomes. **A** Western blot detected the exosome protein biomarkers CD9, CD81, TSG101. **B** and **C** Exosome morphological characteristics and size were determined by transmission electron microscope and dynamic light scattering. **D** Fluorescence microscope was used to trace the CFs exosome location in H/R cardiomyocytes

To further probe the roles of exosomes in suppressing pyroptosis of cardiomyocytes, we first isolated exosomes secreted by CFs. Positive expression of exosome markers, including CD9, CD81, and TSG101, confirmed the successful isolation of exosomes (Fig. 3A). The morphology and size of exosomes were also verified by transmission electron microscope (TEM) and dynamic light scattering analysis (DLS) analysis (Fig. 3B, C). We then fluorescently labeled the isolated exosomes and co-incubated them with cardiomyocytes. As shown Fig. 3D, significant uptake of exosomes in cardiomyocytes was observed (Fig. 3D), suggesting that exosomes secreted by CFs may have biological function in cardiomyocytes.

**Fig. 4:**
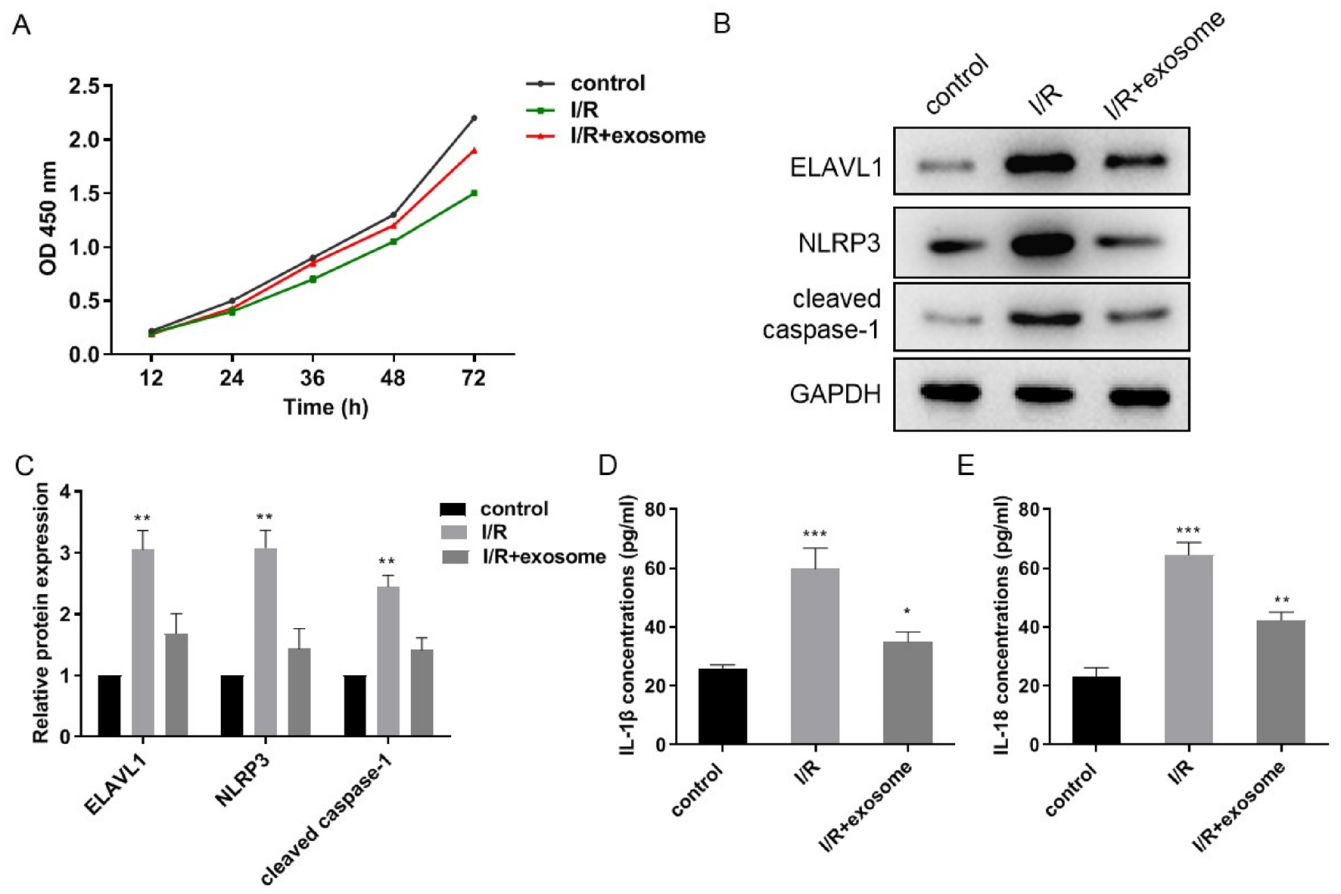
CFs reduce pyroptosis of cardiomyocytes through exosomes. **A** Cell viability was measured by CCK8 kit when exosome was co-cultured with H/R cardiomyocytes. **B** and **C** Western blot analysis the expression of ELAVL1, NLRP3, and cleaved caspase-1 in H/R cardiomyocytes that co-cultured with exosome. **D** and **E** ELISA assay detected the IL-1β, IL-18 protein expression in H/R cardiomyocytes that co-cultured with exosome. *n* = 3, data are shown as mean ± SD. *p < 0.05, **p < 0.01, ***p < 0.001 *vs*. the control group

We then treated cardiomyocytes with H/R or simultaneously with exosomes from CFs. Cell death was first examined. As shown in Fig. 4A, exosomes from CFs remarkably suppressed cardiomyocyte death rates elicited by H/R. We also measured expression levels of pyroptosis markers in treated cardiomyocytes and inflammatory cytokines secreted by cardiomyocytes. Consistent results were obtained, showing that exosomes remarkably decreased the expression of these biomolecules (Fig. 4B–E). Taken together, exosomes secreted by CFs can reduce cardiomyocyte pyroptosis in H/R injury, supporting that cardioprotective function of CFs in MI/R injury is executed through reducing pyroptosis of cardiomyocytes by exosomes.

### Exosome miR-133a targets ELAVL1 in cardiomyocytes

**Fig. 5:**
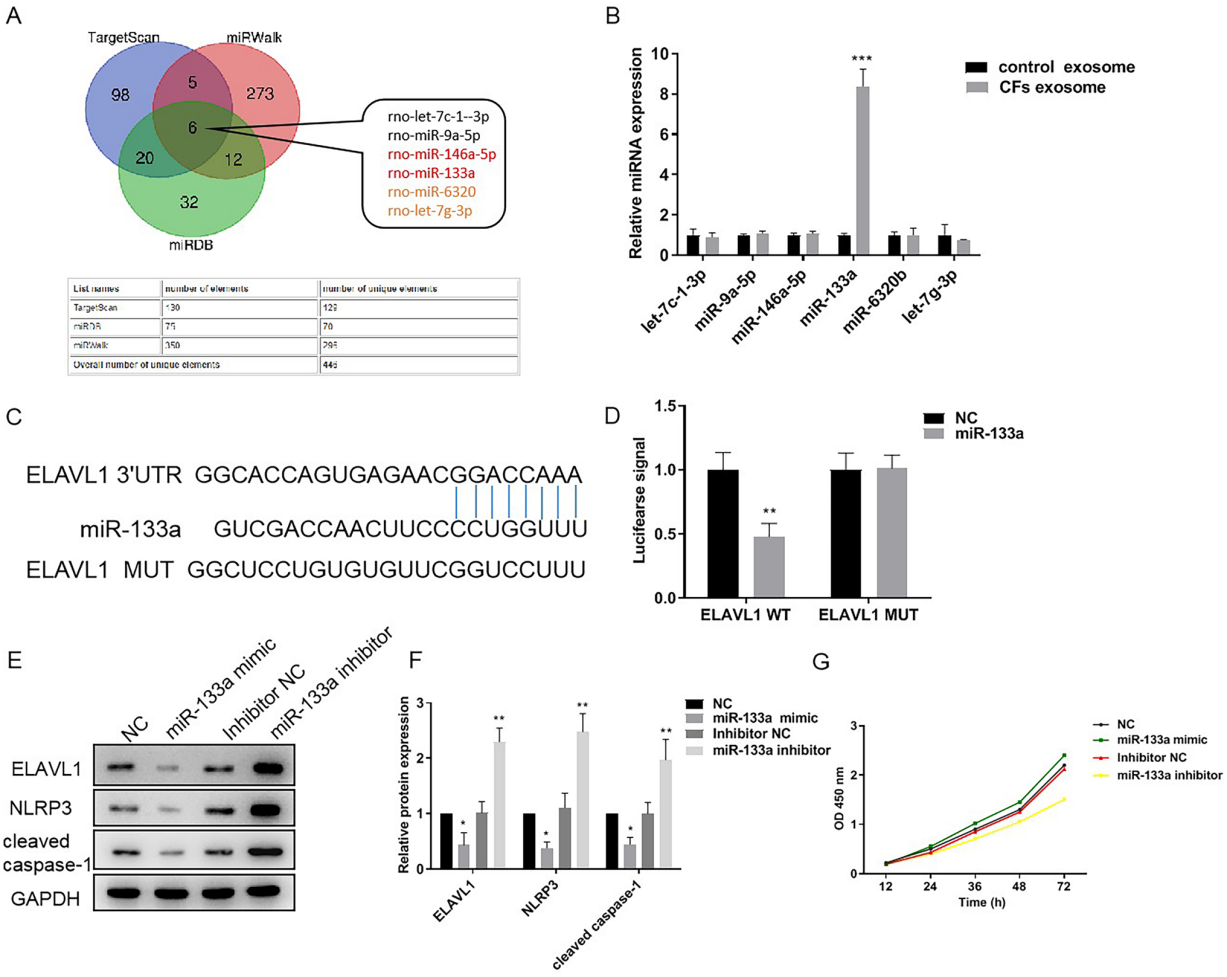
Exosome miR-133a targets ELAVL1 and reduce pyroptosis in cardiomyocytes. **A** Prediction miRNAs target ELAVL1 by TargetScan, miRWalk, and miRDB database. **B** qPCR detected miRNAs expression predicted target binding through three databases in control exosome and CFs exosome. Control exosomes secreted by 293T cells. **C** The putative miR-133a binding sites in 3′UTR of ELAVL1. Replacement of Adenine bases with Uracil (A to U) can be used for the construction of mutant reporter. **D** The relative luciferase activity of cells that were co-transfected with constructed luciferase reporters (ELAVL1 WT or ELAVL1 MUT), and miR-133a mimics or negative control. **E** and **F** The expression of ELAVL1, NLRP3, and cleaved caspase-1 in H/R cardiomyocytes by western blotting when transfected with miR-133a mimics or miR-133a inhibitor. **G** Cell viability was measured by CCK8 kit in H/R cardiomyocytes that transfected with miR-133a mimics or miR-133a inhibitor. *n* = 3, data are shown as mean ± SD. *p < 0.05, **p < 0.01, ***p < 0.001 *vs*. the NC group

To further reveal the underlying molecular mechanism, we then analyzed the contents in exosomes secreted by CFs. As a class of gene regulators, miRNAs can suppress gene expression at the post-transcriptional level [25]. It has been demonstrated that the intercellular communication of CFs with cardiomyocytes can be elicited by exosome miRNAs [18]. However, whether exosome miRNAs can suppress pyroptosis of cardiomyocytes remains underexplored. We focused on the functional consequences of miRNAs in exosomes secreted by CFs. Moreover, since ELAVL1 is a RNA-binding protein that exhibits a pro-inflammatory function [26] and knockdown of ELAVL1 inhibits pyroptosis of cardiomyocytes [23], we continued further investigation with a focus of miRNA-mediated silencing of ELAVL1. It is also worth mentioning that exosomes of CFs indeed down-regulated ELAVL1 protein levels in cardiomyocytes (Fig. 4B, C). We then used computational methods, including TargetScan, miRWalk, and miRDB, to predict miRNAs that can target ELAVL1. As shown in Fig. 5A, we found 6 miRNAs that could be simultaneously predicted by these three methods. We then measured levels of these miRNAs in exosomes secreted by CFs and found that miR-133a was significantly abundant (Fig. 5B). To validate whether miR-133a targets the 3′-untranslated region (3′-UTR) of ELAVL1, we inserted the predicted binding site of miR-133a in ELAVL1 into 3′-UTR of luciferase (Fig. 5C). As a control, we mutated the binding site and constructed a mutant luciferase reporter gene using the same method. Luciferase signals in cardiomyocytes that were co-transfected with luciferase reporter gene and miR-133a or scramble control (NC) were measured, confirming that miR-133a regulated ELAVL1 through binding to its 3’-UTR (Fig. 5D). We further explored the function of miR-133a through transfecting cardiomyocytes with miR-133a mimic or antisense miR-133a. Expression levels of ELAVL1 and other pyroptosis markers were then checked, showing that miR-133a significantly decreased protein levels of ELAVL1, NLPR3, and caspase-1 (Fig. 5E, F). In contrast, knockdown of miR-133a with antisense miR-133a had opposite effects (Fig. 5E, F). Meanwhile, cell death rates were also decreased by miR-133a and increased by antisense miR-133a (Fig. 5G). Taken together, miR-133a in exosomes secreted by CFs can reduce pyroptosis of cardiomyocytes through suppressing ELAVL1.

### Exosome miR-133a protects against MI/R injury in vivo

**Fig. 6:**
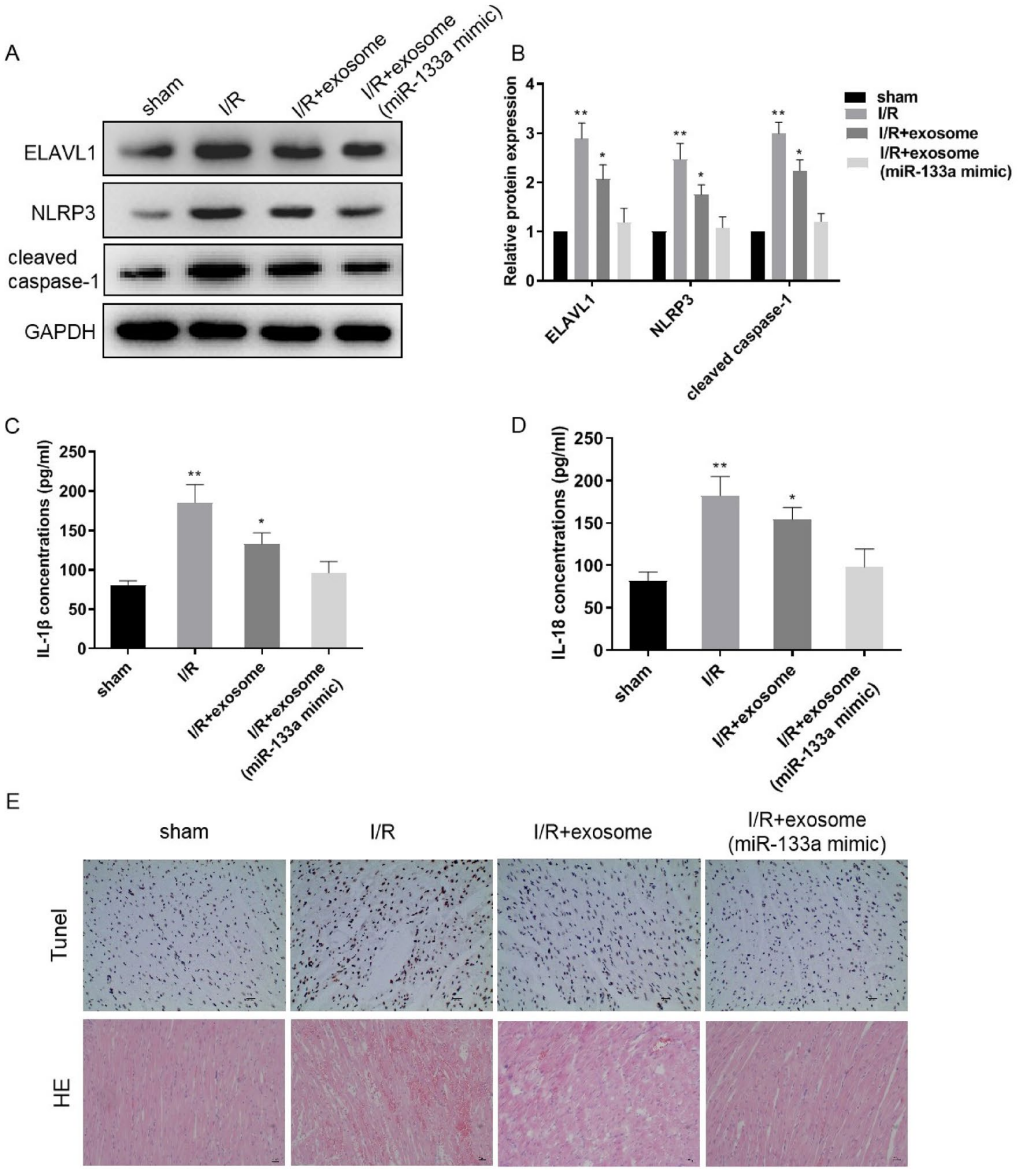
Exosome miR-133a protects against MI/R injury in vivo. **A** and **B** Protein expression and quantitative analysis in rat heart tissues. **C** and **D** ELISA detected the IL-1β, IL-18 expression in rat serum. **E** Histopathology and cell death were analyzed by HE and Tunel staining. *n* = 3, data are shown as mean ± SD. *p < 0.05, **p < 0.01 *vs*. the sham group

Finally, based on these findings, we explored the possibility by using exosome miR-133a to protect MI/R injury in vivo. We developed the MI/R injury rat models and treated them with exosomes secreted from CFs or CFs with miR-133a overexpression. Heart tissues were then collected and expression levels of pyroptosis markers were investigated. As expected, I/R treatment significantly increased ELAVL1, NLRP3, and caspase-1 protein levels (Fig. 6A, B). In comparison, exosomes derived from CFs down-regulated the expression levels of these pyroptosis markers. Notably, exosome with miR-133a overexpression remarkably decreased expressions of these markers. Moreover, the expression levels of inflammatory cytokines in heart tissues were also modulated in a similar pattern (Fig. 6C, D). Cardiomyocyte deaths were aggravated by I/R treatment and significantly reduced by exosomes or exosomes with miR-133a overexpression (Fig. 6E). Taken together, exosomes derived from CFs can reduce pyroptosis of cardiomyocytes and protect MI/R injury in vivo. This effect can be further strengthened by using exosomes from CFs with miR-133a overexpression.

## Discussion

MI/R injury is a life-threatening event that can lead to acute myocardial infarction, heart failure and SCD. Exploration of molecular mechanisms involved in MI/R injury and development of therapeutic strategies to reduce MI/R injury are thus actively pursued. While plenty of efforts have been made, the underlying molecular mechanisms are still not well elucidated and strategies to protect MI/R injury have not shown enough effectiveness in therapeutic treatment [5, 6]. Related studies are thus still highly demanded. In this paper, we reported a novel molecular event during MI/R injury. We found that cardioprotective function of CFs on MI/R injury resulted from suppression of cardiomyocytes pyroptosis.

It has been well demonstrated that cardiomyocytes death, including pyroptosis, is vital and can be a target to protect MI/R injury [7, 8, 13]. Moreover, while CFs have long been known to have protective roles in MI/R injury [16], connection between CFs and cardiomyocytes pyroptosis have not been unveiled. In this paper, we revealed that suppression of cardiomyocyte pyroptosis is a key process for CFs to protect MI/R injury. This phenomenon then drove us to explore the underlying molecular events. For this goal, we focused on exosomes and biomolecules packaged inside that were known to mediate the intercellular communication between CFs and cardiomyocytes [17, 27]. For instance, previous reports indicate that exosomes derived from CFs increase cardiomyocytes survival through delivering tissue inhibitor of metalloproteinases-1 [16, 27]. In our findings, we also discovered that exosomes derived from CFs could suppress pyroptosis and reduce MI/R injury. Delineating the key biomolecules in exosomes of CFs that are involved in this process is thus of great importance to advance both understanding and therapeutic development of MI/R injury.

As endogenous gene silencers, miRNAs have gained much attention, due to their extensive involvement in both physiology and pathology [28]. Previous reports have indicated the important roles of miRNAs in cardiomyocytes and CFs. For instance, during pyroptosis of cardiomyocytes, miRNA-9 that can regulate ELAVL1 to reduce pyroptosis is decreased [23]. For controlling proliferation and migration of CFs, miRNA-19b is found to play pivotal roles through targeting Pten [29]. These pieces of evidence suggest that miRNAs may be involved in suppression of pyroptosis and reducing MI/R injury by exosomes of CFs. Notably, miRNAs are also found to be enriched in exosomes of CFs and exert cardioprotective function [18, 30]. According to our results, we found that miR-133a was enriched in exosomes of CFs and delivered into cardiomyocytes to repress pyroptosis through regulating ELAVL1. This novel mechanism thus provided us a strategy that used exosomes with overexpression of miR-133a to further enhance the therapeutic outcomes of exosomes from CFs. Both caspase-1 and caspase-11 have been shown to be involved in the pyroptosis of cardiomyocytes under H/R condition. Our study showed that caspase-1 is activated by H/R in cardiomyocytes while decreased with the co-incubation of CFs, suggesting caspase-1 activation is important to pyroptosis of cardiomyocytes. In consistence to our data, Zhen Qiu et al also showed the exacerbated MI/R injury in diabetic rats is associate with the upregulated levels of both procaspase-1 and active caspase-1 (Oxid Med Cell Longev. 2017;2017:9743280.). However, a most recent study showed that H/R specifically activates inflammasome through caspase-11 but not caspase-1 in cardiomyocytes. (Circ Res. 2021 Jul 23;129(3):383–396.). The mechanisms underlying the selective action of caspase-1 or caspase-11 is largely unknown but deserve future investigation. Moreover, while we focused on miRNAs encapsulated in exosomes in our study, systematic analysis of exosomes of CFs deserves exploration in the future, since many biomolecules are key orchestrators in MI/R injury [14, 31, 32].

Most of the current studies focused on mesenchymal stem cell (MSC)-exosome that attenuated myocardial injury [33]. However, the relationship between CFs exosome and myocardial injury is largely unknown. In this study, we focused on the CFs exosome effect myocardial cell pyroptosis through miRNAs. Pyroptosis is a form of cardiac cell death that affects MI/R injury. Autophagy and apoptosis are both vital and they can be targets to protect against MI/R injury [34–36]. So whether CFs ameliorate MI/R injury through myocardial autophagy and apoptosis needs to be further explored. Exosome contains a variety of functional molecules, including mRNAs, miRNAs, lncRNAs, and proteins, which is involved in intercellular communication through transferring their genetic contents [37]. We confirmed that exosome from CFs delivers miR-133a into cardiomyocytes to repress pyroptosis. However, whether exosome from CFs delivers other components to participate in MI/R injury needs additional studies. MI/R is a therapeutic approach for reducing disease injury, but also leads to cell death and additional cell dysfunction [38]. Our research showed that CFs suppress myocardial pyroptosis and reduce MI/R injury through exosome miR-133a by suppressing the expression of ELAVL1 in myocardial cells. The results provide a theoretical basis and treatment targets for the protection of MI/R injury. It suggested that using exosomes with overexpression of miR-133a or knockdown of ELAVL1 expression could further enhance the therapeutic outcomes of MI/R injury.

In summary, we have revealed a novel mechanism regarding to the cardioprotective roles of CFs in suppressing cardiomyocytes pyroptosis and ameliorating MI/R injury. The discovery of this new signaling pathway may pave a way to facilitate both understanding and therapeutic treatment of MI/R injury.

## Data Availability

The data that support the findings of this study are available from the corresponding authors on reasonable request.

## References

[1] Roger VL, Go AS, Lloyd-Jones DM, Benjamin EJ, Berry JD, Borden WB, Bravata DM, Dai S, Ford ES, Fox CS, Fullerton HJ, Gillespie C, Hailpern SM, Heit JA, Howard VJ, Kissela BM, Kittner SJ, Lackland DT, Lichtman JH, Lisabeth LD, Makuc DM, Marcus GM, Marelli A, Matchar DB, Moy CS, Mozaffarian D, Mussolino ME, Nichol G, Paynter NP, Soliman EZ, Sorlie PD, Sotoodehnia N, Turan TN, Virani SS, Wong ND, Woo D, Turner MB, American Heart Association Statistics C and Stroke Statistics S (2012). Heart disease and stroke statistics–2012 update: a report from the American Heart Association. Circulation.

[2] Anderson JL, Morrow DA (2017). Acute myocardial infarction. N Engl J Med.

[3] Yellon DM, Hausenloy DJ (2007). Myocardial reperfusion injury. N Engl J Med.

[4] Chen J, Luo Y, Wang S, Zhu H, Li D (2019). Roles and mechanisms of SUMOylation on key proteins in myocardial ischemia/reperfusion injury. J Mol Cell Cardiol.

[5] Altamirano F, Wang ZV, Hill JA (2015). Cardioprotection in ischaemia-reperfusion injury: novel mechanisms and clinical translation. J Physiol.

[6] Davidson SM, Ferdinandy P, Andreadou I, Botker HE, Heusch G, Ibanez B, Ovize M, Schulz R, Yellon DM, Hausenloy DJ, Garcia-Dorado D, Action CC (2019). Multitarget strategies to reduce myocardial ischemia/reperfusion injury: JACC review topic of the week. J Am Coll Cardiol.

[7] Del Re DP, Amgalan D, Linkermann A, Liu Q, Kitsis RN (2019). Fundamental mechanisms of regulated cell death and implications for heart disease. Physiol Rev.

[8] Jia C, Chen H, Zhang J, Zhou K, Zhuge Y, Niu C, Qiu J, Rong X, Shi Z, Xiao J, Shi Y, Chu M (2019). Role of pyroptosis in cardiovascular diseases. Int Immunopharmacol.

[9] Aizawa S, Brar G, Tsukamoto H (2020). Cell death and liver disease. Gut Liver.

[10] Bian Y, Li X, Pang P, Hu XL, Yu ST, Liu YN, Li X, Wang N, Wang JH, Xiao W, Du WJ, Yang BF (2020). Kanglexin, a novel anthraquinone compound, protects against myocardial ischemic injury in mice by suppressing NLRP3 and pyroptosis. Acta Pharmacol Sin.

[11] Toldo S, Mauro AG, Cutter Z, Abbate A (2018). Inflammasome, pyroptosis, and cytokines in myocardial ischemia-reperfusion injury. Am J Physiol Heart Circ Physiol.

[12] Qiu Z, Lei S, Zhao B, Wu Y, Su W, Liu M, Meng Q, Zhou B, Leng Y, Xia ZY (2017). NLRP3 Inflammasome activation-mediated Pyroptosis aggravates myocardial ischemia/reperfusion injury in diabetic rats. Oxid Med Cell Longev.

[13] Rauf A, Shah M, Yellon DM, Davidson SM (2019). Role of caspase 1 in ischemia/reperfusion injury of the myocardium. J Cardiovasc Pharmacol.

[14] Nazir S, Gadi I, Al-Dabet MM, Elwakiel A, Kohli S, Ghosh S, Manoharan J, Ranjan S, Bock F, Braun-Dullaeus RC, Esmon CT, Huber TB, Camerer E, Dockendorff C, Griffin JH, Isermann B, Shahzad K (2017). Cytoprotective activated protein C averts Nlrp3 inflammasome-induced ischemia-reperfusion injury via mTORC1 inhibition. Blood.

[15] Chen W, Frangogiannis NG (2013). Fibroblasts in post-infarction inflammation and cardiac repair. Biochim Biophys Acta.

[16] Abrial M, Da Silva CC, Pillot B, Augeul L, Ivanes F, Teixeira G, Cartier R, Angoulvant D, Ovize M, Ferrera R (2014). Cardiac fibroblasts protect cardiomyocytes against lethal ischemia-reperfusion injury. J Mol Cell Cardiol.

[17] Vanhaverbeke M, Gal D, Holvoet P (2017). Functional role of cardiovascular exosomes in myocardial injury and atherosclerosis. Adv Exp Med Biol.

[18] Wendt S, Goetzenich A, Goettsch C, Stoppe C, Bleilevens C, Kraemer S, Benstoem C (2018). Evaluation of the cardioprotective potential of extracellular vesicles—a systematic review and meta-analysis. Sci Rep.

[19] Burke RM, Lighthouse JK, Quijada P, Dirkx RA, Rosenberg A, Moravec CS, Alexis JD, Small EM (2018). Small proline-rich protein 2B drives stress-dependent p53 degradation and fibroblast proliferation in heart failure. Proc Natl Acad Sci USA.

[20] Thery C, Amigorena S, Raposo G, Clayton A (2006). Isolation and characterization of exosomes from cell culture supernatants and biological fluids. Curr Protoc Cell Biol.

[21] Maroto R, Zhao Y, Jamaluddin M, Popov VL, Wang H, Kalubowilage M, Zhang Y, Luisi J, Sun H, Culbertson CT, Bossmann SH, Motamedi M, Brasier AR (2017). Effects of storage temperature on airway exosome integrity for diagnostic and functional analyses. J Extracell Vesicles.

[22] Hampton CR, Shimamoto A, Rothnie CL, Griscavage-Ennis J, Chong A, Dix DJ, Verrier ED, Pohlman TH (2003). HSP70.1 and -70.3 are required for late-phase protection induced by ischemic preconditioning of mouse hearts. Am J Physiol Heart Circ Physiol.

[23] Jeyabal P, Thandavarayan RA, Joladarashi D, Suresh Babu S, Krishnamurthy S, Bhimaraj A, Youker KA, Kishore R, Krishnamurthy P (2016). MicroRNA-9 inhibits hyperglycemia-induced pyroptosis in human ventricular cardiomyocytes by targeting ELAVL1. Biochem Biophys Res Commun.

[24] Maring JA, Beez CM, Falk V, Seifert M, Stamm C (2017). Myocardial regeneration via progenitor cell-derived exosomes. Stem Cells Int.

[25] Bartel DP (2018). Metazoan microRNAs. Cell.

[26] Krishnamurthy P, Lambers E, Verma S, Thorne T, Qin G, Losordo DW, Kishore R (2010). Myocardial knockdown of mRNA-stabilizing protein HuR attenuates post-MI inflammatory response and left ventricular dysfunction in IL-10-null mice. FASEB J.

[27] Giricz Z, Varga ZV, Baranyai T, Sipos P, Paloczi K, Kittel A, Buzas EI, Ferdinandy P (2014). Cardioprotection by remote ischemic preconditioning of the rat heart is mediated by extracellular vesicles. J Mol Cell Cardiol.

[28] Rupaimoole R, Slack FJ (2017). MicroRNA therapeutics: towards a new era for the management of cancer and other diseases. Nat Rev Drug Discov.

[29] Zhong C, Wang K, Liu Y, Lv D, Zheng B, Zhou Q, Sun Q, Chen P, Ding S, Xu Y, Huang H (2016). miR-19b controls cardiac fibroblast proliferation and migration. J Cell Mol Med.

[30] Bang C, Batkai S, Dangwal S, Gupta SK, Foinquinos A, Holzmann A, Just A, Remke J, Zimmer K, Zeug A, Ponimaskin E, Schmiedl A, Yin X, Mayr M, Halder R, Fischer A, Engelhardt S, Wei Y, Schober A, Fiedler J, Thum T (2014). Cardiac fibroblast-derived microRNA passenger strand-enriched exosomes mediate cardiomyocyte hypertrophy. J Clin Invest.

[31] Woodall MC, Woodall BP, Gao E, Yuan A, Koch WJ (2016). Cardiac fibroblast GRK2 deletion enhances contractility and remodeling following ischemia/reperfusion injury. Circ Res.

[32] Qin CX, May LT, Li R, Cao N, Rosli S, Deo M, Alexander AE, Horlock D, Bourke JE, Yang YH, Stewart AG, Kaye DM, Du XJ, Sexton PM, Christopoulos A, Gao XM, Ritchie RH (2017). Small-molecule-biased formyl peptide receptor agonist compound 17b protects against myocardial ischaemia-reperfusion injury in mice. Nat Commun.

[33] Zou L, Ma X, Lin S, Wu B, Chen Y, Peng C (2019). Bone marrow mesenchymal stem cell-derived exosomes protect against myocardial infarction by promoting autophagy. Exp Ther Med.

[34] Hu S, Cao S, Tong Z, Liu J (2018). FGF21 protects myocardial ischemia-reperfusion injury through reduction of miR-145-mediated autophagy. Am J Transl Res.

[35] Liu SD, Meng WX, Xu L, Chi C, Sun X, Liu HY (2018). GAS5 promotes myocardial apoptosis in myocardial ischemia-reperfusion injury via upregulating LAS1 expression. Eur Rev Med Pharmacol Sci.

[36] Li Z, Zhang Y, Ding N, Zhao Y, Ye Z, Shen L, Yi H, Zhu Y (2019). Inhibition of lncRNA XIST improves myocardial I/R injury by targeting miR-133a through inhibition of autophagy and regulation of SOCS2. Mol Ther Nucleic Acids.

[37] Mathivanan S, Ji H, Simpson RJ (2010). Exosomes: extracellular organelles important in intercellular communication. J Proteomics.

[38] Kalogeris T, Baines CP, Krenz M, Korthuis RJ (2016). Ischemia/reperfusion. Compr Physiol.

